# Against Symmetry Fundamentalism

**DOI:** 10.1007/s10670-023-00774-4

**Published:** 2024-01-27

**Authors:** Cristian Lopez

**Affiliations:** Université de Lausanne, Lausanne, Switzerland

**Keywords:** Symmetry, Realism, Idealizations, Indispensability, Structures, Laws of Nature, Stipulations

## Abstract

Symmetry fundamentalism claims that symmetries should be taken metaphysically seriously as part of the fundamental ontology. The main aim of this paper is to bring some novel objections against this view. I make two points. The first places symmetry fundamentalism within a broader network of philosophical commitments. I claim that symmetry fundamentalism entails idealization realism which, in turn, entails the reification of further theoretical structures. This might lead to an overloaded ontology as well as open the way to criticisms from metaphysical frameworks that reject such reifications. The second point contrasts symmetry fundamentalism with the now common view that regards symmetries as stipulations guiding empirical research and theory construction. I claim that both views clash each other and cannot be held together. I finish the paper with a more positive prospect that will be developed in future work—symmetry deflationism.

## Introduction

Stable regularities are to be found by eliminating or abstracting away many features that intervene in the unfolding behavior of phenomena as commonly experimented. Only by doing so, can we get at covering (or general) and phenomenological laws, and stable models to explain and predict phenomena. And we can only do this by relying on various theoretical tools and resources scientists have been working with for, at least, the last four centuries. No doubt that symmetries have been one of these central resources not only for explanatory purposes, but also for guiding empirical research and theory construction. New laws, new interactions, new properties and entities have “come out” from symmetry-based arguments. To do physics as we know it, philosophers as well as physicists readily acknowledge symmetries’ theoretical relevance and, going even further, their ineliminable role.

The astonishing success of symmetry-based arguments in physics has in the last decades fed the idea that symmetries are more than theoretical resources. Physicists and philosophers have lately entertained the idea that symmetries might also be aspects of the fundamental reality. Here, physicists’ and metaphysicians’ work seem to intersect each other—if metaphysicians are in the business of discovering which entities, properties, relations, or structures are fundamental in the world, physicists have proposed symmetries as promising candidates to dwell in the world’s fundamental ontology. This view, which I call *symmetry fundamentalism* (**SF** henceforth), following David Schroeren’s work, has been glossed differently but basically amounts to taking symmetries as metaphysically fundamental. Strong versions of **SF** have been advocated by, for instance, Werner Heisenberg (1975), Steven Weinberg (1987), David Baker (2010), Steven French (2014), David Schroeren (2020), Alyssa Ney (2021), among others.

In general, this paper tries to rectify two failures I identify in the philosophical literature on symmetries. First, most analyses have been largely carried out as if symmetries were isolated, unconnected from the rest of elements we find in science (e.g., laws of nature) or from various ways to assess ontological commitments (e.g., parsimony). To my mind, our philosophical interpretation of symmetries must not only be assessed in relation to the rest of all our epistemic and ontological assumptions, but also according to traditional standards to assess ontological commitments. Second, symmetries’ metaphysical fundamentality seems to derive from their indispensability, but I think it is not necessarily so—we can accept that symmetries are indispensable, but not *metaphysically* fundamental. Then, we can resist that symmetries’ indispensability requires us to take them as metaphysically fundamental. A dose of caution is then called for in distinguishing cases in which indispensability demands a metaphysical commitment from cases in which indispensability demands an epistemic commitment.

Under this general approach, this paper raises some novel objections against** SF**. In Sect. 1, I provide a quick and general overview of symmetries in physics. In Sect. 2, I expose some of the different philosophical positions that can be adopted towards symmetries. In Sect. 3, I develop my objections against **SF**: the first one relates to the role of idealizations in our philosophical construal of symmetries in physics. As I argue, **SF** requires to be realist about idealization products and the reification of further theoretical structures as support. The second contrasts **SF** with the now common view that regards many relevant symmetries as stipulations guiding empirical research and theory construction. As I argue, **SF** cannot accommodate symmetries, if they are stipulated. Though this paper is mostly critical, it is meant to pave the way for an alternative view of symmetries’ indispensability that entails no strong realist commitments. *Symmetry deflationism*, as I call it, is briefly advanced in the conclusions and will be explored further in future work.

## Symmetries in Physics (a Quick Overview)

Although physical symmetries come in many flavors and shapes (internal vs. external, local vs. global, theoretical vs. observational, geometrical vs. dynamical, and so on), all of them are for the most part *formal* notions that apply to mathematical structures. From a general perspective, physical symmetries are transformations that keep some relevant structure unaltered. In physics, most mathematical structures of interest are sets of differential equations that relate to other mathematical structures (e.g., topological and differential spaces). In consequence, physical symmetries are transformations that preserve the space of solutions of such sets of differential equations. In this precise sense, physical symmetries are said to be structure-preserving functions that map solutions to solutions over a well-defined space. This is the *formal* definition of a physical symmetry. In a classical setting and in the group-theorical language, it can be defined as follows (see Olver, 1993: 93; see also Belot, 2013).

**Formal**_**def**_: Suppose a system $\Delta$ of differential equations involving p independent variables ($x={x}^{1}\dots {x}^{p})$ and q dependent variables ($u={u}^{1}\dots {u}^{q})$. The solutions of $\Delta$ are of the form $u=f(x)$. Let $X={\mathbb{R}}^{p}$, with coordinates $x={x}^{1}\dots {x}^{p}$, be the space representing the independent variables, and let $U={\mathbb{R}}^{q}$, with coordinates $u={u}^{1}\dots {u}^{q}$, represent the dependent variables. A classical symmetry group of the system $\Delta$ will be a local group of transformations, $G$, acting on some open subset $M\subset X\times U$ (kinematically possible fields) in such a way that $G$ transforms solutions of $\Delta$ to other solutions of $\Delta$.

Of course, not any transformation will count as a *physical* symmetry. If this were so, the concept would be trivial and it would always be possible to define a transformation that maps solutions to solutions, augmenting the symmetries of a theory at demand. As Gordon Belot mentions (2013), symmetries are rather hard to come by, so their physical definition should be not too liberal. This in general amounts to imposing further constraints on the formal definition. Some of them can be also purely formal—e.g., for Lie transformations, they must be continuous or smooth; for classical symmetries, the infinitesimal generators must depend only on the independent and dependent variables of the theory, that is, their functions describe infinitesimal variations only for the independent (e.g., time and spatial coordinates) and dependent variables (e.g., dynamical variables); in the case of more general symmetries, for instance, the infinitesimal generators might depend not only on the independent and dependent variables, but also on the derivatives of the fields (Lie-Bäcklund transformations). Others can be physical—e.g., Hamiltonian symmetries are required to not only preserve the geometrical structure of the phase space, but also the Hamiltonian. And others can be interpretative—e.g., physical symmetries are required to preserve the observational content of a physical theory (see, for instance, Roberts 2008, Dasgupta, 2016), or to identify surplus structure (see Redhead, 1975, Dewar 2019).

I acknowledge that it is an open question in the literature which interpretative constraint is the adequate one to provide a satisfactory definition of physical symmetries and I do not intend to settle the discussion here (for in detail discussions of the different views, see Dasgupta 2016, Wallace 2019). For practical purposes and mutual understanding, I say that a physical symmetry is:

**Physical symmetry**_**def**_: A structure-preserving transformation acting upon a set of differential equations such that it (a) satisfies the formal definition, (b) keeps invariant physically relevant objects of the theory (e.g., the Hamiltonian, the Lagrangian, etc.), and (c) fulfils some interpretative constraint.

For instance, if (c) refers to the preservation of observational content, a physical symmetry will preserve the observational sentences that an agent would report when asked how things appear in two symmetry-related scenarios. This yields the concept of ‘observational equivalence’ such that two symmetry-related models must be also observationally equivalent.

Since symmetries in this robust sense are not easy to come by, they are generally found after a laborious process of formalization and idealization. When a physical theory (or a law) is claimed to possess a symmetry, what is generally meant is that some specific expression of its equations of motion (i.e., the “general law” or “covering law”, see Dray, 1957, Hempel, 1965, and Cartwright, 1983) exhibits the symmetry. It immediately translates into the kind of behavior that idealized entities exhibit in a model (as a conceptual object).^1^ The remark is noteworthy because a vast number of instantiations of the covering laws and more phenomenological models won’t surely exhibit the symmetry at issue. Think of simple space–time symmetries as spatial rotations—only by abstracting away most interactions and forces we may get at general enough formulations of the laws (and, consequently, some models) that exhibit the symmetry. Therefore, symmetries generally require us to also draw our attention to very specific structures, and not only to meet the above-mentioned definition.

There are at least three kinds of symmetries that have specially drawn philosophers’ attention in the last decades—*space–time* symmetries, *permutation* symmetries, and *local gauge* symmetries. Although permutation symmetries have been central in quantum statistics and would very well fit into a **SF**’s framework (see French, 2014), for simplicity I will set them aside and focus on space–time (or geometric) symmetries and local gauge symmetries.

Space–time symmetries play a structuring role in theory construction since they set the geometrical setting over which the dynamics will unfold. This has suggested a remarkable connection between “law symmetries” and “space–time symmetries”—desirably, any space–time symmetry is a dynamical symmetry, and vice versa (see Earman, 1989: 46).^2^ This insight has been incredibly powerful. For instance, that the equations of motion of a physical theory are space-translation invariant means that the spatial structure giving support to such laws must be homogeneous, that is, that there must not be privileged spatial point on the manifold. In physical terms, it means that the behavior of a physical system must not depend on the position the system is at.

This “principle of adequacy” should however be generalized. The matching between the symmetries of the dynamics and the symmetries of the supporting geometrical structure does not circumscribe to space–time structures, but also extends to other geometries and types of symmetries. Those who are realist about configuration space (Albert, 1996; North, 2013) or state space (Schroeren, 2020) will presumably regard the principle as correlating the symmetries of the dynamics with the symmetries of the geometry of such spaces. In the case of local gauge symmetries, a general symmetry principle can be also obtained: every kinematical symmetry of a theory should also be a dynamical symmetry, and vice versa (see Hetzroni 2020, also Wigner 1964) It is worth noting the *normative* nature of the principle—any symmetry at the level of the dynamics *should be* a symmetry at the level of the supporting geometry (or the kinematics), and vice versa. They serve as norms to formulate appropriate physical theories, allowing us to correlate two at-first-glance different types of symmetries.

Finally, the canonical way to understand fundamental interactions (electroweak and strong interactions) in particle physics is via the so-called “gauge paradigm”. The demand of local gauge symmetries has been crucial in formulating field theories and modelling interactions in particle physics. In essence, local gauge symmetries “localize” global symmetries (e.g., global phase transformation) by making the transformation (e.g., $\Lambda )$ depend on the coordinates (e.g., $\Lambda ({\text{x}})$). In demanding local gauge symmetry, the Lagrangian needs to be modified and new compensatory fields introduced (the gauge potential). The “gauge argument”, as this rationale has been called (Martin, 2002), dictates new interactions (coupling) between matter and the gauge field, which have not only led to astonishingly successful physics in the last fifty years but would also purportedly reveal a deep “logic of nature” (Martin, 2003). New interactions and new fields seem to be “dictated” by local gauge symmetries.

## Indispensability, Realism and Fundamentality

Physics presents us with symmetries. This is an easily verified fact—modern physics’ discourse involves propositions ${p}_{i}$ stating that in a theoretical framework a symmetry $\sigma$ is the case. For lack of a better expression, I call this “**the symmetry fact**”. It also happens that **the symmetry fact** has led to successful physics (as well as the formulation of simple laws has led to successful physics). Symmetries have been crucial in *physical explanations* (e.g., spontaneous symmetry breaking in solid state), in *empirical research* (e.g., the discovery of new particles, as the negative omega baryon, ${\Omega }^{-}$, by Barnes and colleagues in 1964), and in *theory construction*, playing a structuring role (e.g., a theory’s symmetry group, like the Galilei Group in non-relativistic standard quantum mechanics or Bohmian mechanics, see Ballentine 1998, Dürr & Teufel, 2009).

I believe that **the symmetry fact,** that is, the existence of such propositions, is undeniable. What’s interesting though is what philosophical attitude we may take towards it. Some may claim that **the symmetry fact** shows that symmetries are fundamental. In the philosophical field, the word ‘fundamental’ might yet be controversial. On the one hand, one might want to distinguish between what’s fundamental in reality and what’s fundamental in our way of representing reality. This is however non-standard—in metaphysics, what is labeled as ‘fundamental’ is also *metaphysically* fundamental, that is, a relation, an entity or a property that is part of the basic ontology the rest of reality is made of. To avoid any confusion, I will rather refer to symmetries as indispensable, and as *symmetry indispensabilism* (**SInd**) the view that holds that for various theoretical and empirical reasons, the propositions in **the symmetry fact** must be taken to be indispensable in physics theorizing. By ‘indispensable’ here I mean that we cannot do physics as we do it without propositions involving symmetry claims; consequently, we should not expect to eliminate them in the future (contrast this, in physics, with Hossenfelder 2018 and, in philosophy, with Bird, 2007: 214). This position is quite well represented, for instance, by Joe Rosen –we *understand* nature in the language of symmetry and science is *founded* in symmetries (2008: ix, 17).

Note that **SInd** remains silent about why propositions involving symmetries cannot be eliminated. In fact, we could mean that symmetries are indispensable because either they, somehow, refer to some (fundamental) aspect of physical reality, or they serve some highly valued epistemic purpose in physics theorizing. While both views can equally embrace **SInd**, they do it in two different directions—the former grounds **SInd** in what the world is like, whereas the latter in our means to represent reality. This distinction is noteworthy because, in the current literature, it is frequently overlooked. The indispensable role of symmetries (as in Heisenberg, 1975, Weinberg & Feynman, 1987, French, 2014, or Schroeren, 2020) is sometimes meant as explicitly expressing (or directly entailing) not only some realist commitment to symmetries, but also their fundamentality, while its rejection might misleadingly suggest that we are committed to taking symmetries as dispensable. My definition of **SInd** is neutral since it is compatible with metaphysical and epistemic attitudes towards symmetries.

**SInd** may lead to some form of symmetry realism. But it is the thesis that symmetries are not only real, but also fundamental, what leads to *symmetry fundamentalism* (**SF**). One way to view **SF** is that it justifies **SInd** on a metaphysical basis—symmetries are indispensable because they are (or directly represent) aspects of the fundamental ontology. By “fundamental ontology” I mean a “complete minimal basis” (Tahko, 2014: 263), that is the set of entities, properties, relations and/or process out of which the rest of the ontology can be obtained; they are, so to speak, the “building blocks” that determine everything else (see also Schaffer, 2010). **SF** can then be viewed as **SInd** plus a strong realist attitude towards symmetries and their promotion as part of the basic entities, properties or relations that compose a complete minimal basis:

**SF**: All the putative references to symmetries appearing in the **symmetry fact** refer to entities, properties, or relations of the world’s fundamental ontology

It just happens that the world has such a structure that makes our theoretical statements involving symmetries (approximately) true, which is just a wordy way to say that symmetries are indispensable because they are (somehow) part of the fundamental reality. Three arguments uphold **SF** as stated—a type of the No Miracle argument, a type of the Quine-Putnam Indispensability argument, and some relation of ontological dependence between symmetries and the rest of our ontology. According to the former, from the **symmetry fact** we can infer that the empirical success of the introduction of symmetries in physics can only be explained by stating that symmetries must be ‘out there’ in the world; otherwise, the empirical success of modern physics would be a miracle. Since we do not believe in miracles, the best explanation of the **symmetry fact** is that symmetries must be ‘out there’ in the world. A No Miracle type argument therefore forces us to take symmetries as real, supporting some form of symmetry realism. But note that this is a necessary, though not sufficient condition for **SF**: that something is real does not per se mean that it also belongs to the common minimal basis that determines everything else.

A type of Quine-Putnam Indispensability Argument complements a No Miracle argument and places symmetries indispensably in one’s ontology^3^:

P1. We ought to be ontologically committed to only those theoretical posits that are indispensable to our (best) physical theories,

P2. Symmetries are properties of dynamical equations that are indispensable to our best physical theories,

C. Therefore, we ought to be ontologically committed to symmetries.

There seems to be some immediate step from this argument to the claim that symmetries are therefore metaphysically fundamental—after all, if they are indispensable and we are then ontologically committed to them, but they are however not metaphysically fundamental, they therefore seem to enter the ontology as an ‘ontological free-lunch’ (to employ Armstrong’s expression, see Armstrong 1997: 12, Schaffer, 2009: 353). It would declare them as derivative entities that are “no addition to being” and, therefore, dispensable in an ontological sense.^4^ This just contends the starting point of the argument, undermining **SF** altogether. In any case, it might be useful to add an argument to back the idea that symmetries are not merely part of one’s ontology (which merely delivers us a form of symmetry realism), but part of one’s *fundamental* ontology. When the **symmetry fact** is closely examined, symmetries does not merely ‘appear’ in our physical theories, but they also play a (theoretical) grounding role—for instance, a theory’s dynamics is seen as relying on symmetry statements; symmetries are said to ‘dictate’ the fundamental interactions or, even, the kind of particle that intervene. So, it is very plausible to read this symmetry-way-of-talking as representing an ontological dependence relation as well. In the theory, it has been argued, symmetry facts ground, for instance, nomological facts or property facts (e.g., facts about elementary particle masses or energies). On this basis, there is also an ontological relation that place symmetries as ontologically fundamental, and the rest as derivative or supervenient (see, for instance, McKenzie, 2014; for a call for caution, see Brading & Brown, 2003). I want to remain silent about which specific relation may connect the fundamental ontology with the derivative ontology(supervenience, grounding, reduction, emergence, etc.), but I do believe that **SF** requires a metaphysical relation that connects the common minimal basis with everything else.

**SF** counts on many venerable advocates. In the physicist camp, Steven Weinberg (1987, 1993), Abdus Salam (1989), Richard Feynman (1987) and even Werner Heisenberg (1975) have held some form of **SF**. Just to provide an example, Weinberg and Feynman famously claimed that “symmetries are fundamental, and possibly all that one needs to learn about the physical world beyond quantum mechanics itself” (Weinberg & Feynman, 1987: 79). In the philosophy camp, **SF** has lately taken many forms. One version relies on Ontic Structural Realism, yielding Group Structural Realism (French, 2014). According to it, the symmetries and the laws of physical theories give us the features of the structure of the world, in contraposition to an object-oriented metaphysics where the laws and the symmetries are underpinned by property-possessing objects (French, 2014: ix). David Schroeren (2020, 2021) has overtly defended **SF**.^5^ For him, symmetries are fundamental aspects of physical reality (‘building blocks’) in which everything else (fields, particles, macroscopic objects) is grounded.

To see some examples, Steven French (2014) says:

“Here the difference between the object-oriented and the structural realist comes into play: the former reads her ontology off theories at some remove, by taking the laws and symmetries that the theories present to be underpinned by property-possessing objects to which we should be ontologically committed. The latter reads her ontology off these theories directly, by taking the very same laws and symmetries as features of the structure of the world” (2014: ix)

It is important to note that, according to Group Structural Realism, structures are fundamental, and we should regard symmetries and laws as part of the fundamental structures. In a recent paper, David Schroeren (2020) expresses a similar point:

“Symmetries are fundamental aspects of physical reality, whereas the physical entities that we would ordinarily have thought of as the fundamental building blocks of the physical world—such as elementary particles or fields—are ontologically derivative of these aspects” (2020: 308-309)

It is worth distinguishing between **SF** (or symmetry realism more generally) from another common view that takes symmetries as inputs in philosophical inferences to shape metaphysics. Many philosophers, such as Jill North, Shamik Dasgupta, among others, have argued that symmetries may work as a guide to metaphysics, since they allow philosophers to infer which are the fundamental structures (or properties, or entities) of the world. It is clear that, according to this view, symmetries are *not* part of the fundamental ontology, but they work as guides to the fundamental ontology^6^ (see, for instance, North, 2008, 2013, 2021; Dasgupta 2015).

To sum up, **SInd** is a reasonable attitude towards symmetries in modern physics. However, it does not directly imply a realist commitment to symmetries. If a realist commitment towards symmetries is also endorsed and symmetries are promoted to structures or elements of the fundamental ontology, we obtain **SF**. Therefore, the rejection of **SF** would not necessarily imply that symmetries may be dispensable, but that their indispensability may be epistemic. I will come back to this in the conclusions. From now on, I will focus on **SF**.

## Two Issues for SF. A Call for Philosophical Caution

I do not mean **SF** to be wrongheaded or incoherent. Indeed, I do think it is a workable philosophical attitude towards symmetries. In the right framework, it makes perfect sense. The point I want to raise concerns such a framework along with **SF**’s implications. More specifically, my concerns should be better viewed as a call for philosophical caution. The main problem is that **SF** prima facie clashes with two widely shared views on symmetries, or at least the most relevant ones:

- they are *idealizations*,
- they are *stipulated* principles.

So, two problems against **SF** will be invoked as support for this call.

First, it is not so straightforward to endorse **SF** about symmetries if they are idealizations (or idealized products)—to take symmetries as metaphysically fundamental does not come free of philosophical implications, but it entails a series of ontological and epistemic commitments in a broader network. In particular, **SF** entails not only symmetry realism, but also a form of *idealization realism* (IR henceforth), that is, the view that takes idealizations as somehow “latching onto” the external world. IR in turn requires to reify further theoretical structures (e.g., the dynamical structure) as the truth-makers of idealizations and, ultimately, as the symmetry bearers. But this raises two worries. First, a rejection of any of **SF**’s implications entails a rejection of **SF**. Second, **SF** might lead to an overloaded (non-parsimonious) ontology if an alternative view of symmetries can be put forward.

The second problem relates to the common view that regards symmetries as principles guiding theory construction and empirical research. I argue that such a view is better construed as implying that many of the most relevant symmetries in physics and in philosophy work as stipulations. **SF** would imply that such stipulations are not merely epistemic resources, but they mysteriously also reveal a deep “logic of nature”. However, it is not clear how this is supposed to work since stipulations may have different roles (e.g., regulative, constitutive, pragmatic, etc.), but none of them directly linked to falsity or truthfulness. This argument does not necessarily assume that *all* symmetries are stipulated or must be stipulated. It might be the case that some relevant symmetries are not stipulated and, in consequence, are not affected by this argument. Notwithstanding this, I submit that most relevant symmetries for philosophers do seem to be stipulated (e.g., space–time symmetries). In consequence, the argument should be read in a conditional form: *if* a symmetry is stipulated, *then* it does not have to do with truthfulness or falsity. Whether a symmetry is stipulated or not must be decided independently.

### First Problem. *Symmetries, Realism and Idealizations*

Imagine that the actual world is Newtonian. In that world, most phenomena are irreversible, in the sense that their behavior is due to many non-conservative forces (e.g., drag forces, friction, etc.). It happens that most instantiations of the (covering) Newtonian dynamics are then non-time-reversal invariant (see Hutchison, 1993)—they are bound to be awfully complicated instantiations of Newton’s second and third laws involving imperfect springs, drag forces, friction, and so on. In adopting **SF**, we might be tempted to quickly jump to the conclusion that there is something metaphysically meaningful about time’s direction here. If symmetries are fundamental aspects of reality, a violation of a symmetry might well be an aspect that reality lacks. After all, a violation of time-reversal invariance would speak in favor of an intrinsic anisotropy of time (see, for instance, Horwich, 1987).^7^

Yet, we are told, the temptation must be resisted—in order to assess the metaphysical status of symmetries, we should not look at phenomenological laws, but at the covering (or general) laws (see Callender, 1995; French, 2014). The argument is that whereas such general equations are *fundamental*, their instantiations are derivative. As Craig Callender (1995) holds, the covering (general) equations capture reality at bottom because they latch onto the fundamental ontology (where there are only conservative forces); the phenomenological laws are rather derivative since they feature non-fundamental (or even unreal!) forces (see Callender, 1995: 333). Then, the symmetries of the covering laws are also aspects of the fundamental reality. This is not a trivial assumption, since it draws a metaphysical line between phenomenological and covering laws; and this assumption is not only at the core of **SF**, but it has also become a received view of how we should assess symmetry claims in physics and philosophy.

It is a fact that what goes by Newtonian mechanics comprehends a huge number of instantiations of Newtonian covering laws. And it turns out that most of these instantiations will be asymmetric under many symmetry transformations of interest (like time reversal, space translation or Galilei boost), whereas the covering laws (the general expressions of the laws) will rather be symmetric.^8^ And it happens that the former vastly outnumber the latter. This is not up to us, but it is due to formal relations that hold in the formulation of classical dynamical laws. What is interesting though is the procedure whereby we reach these general enough expressions of the laws along with the models they aim to describe. In one way or another, it requires us to introduce “distortions” or “falsehoods” in the physical theories. That is, they require a thorough work of simplifying, abstracting away and postulating ideal entities. In a nutshell, it requires *idealizations.*^9^

So, philosophically relevant discussions about symmetries generally depend on drawing our attention towards covering laws. We do not take their instances (as those involving non-conservative forces in Newtonian classical mechanics) to draw metaphysical conclusions about time or space. **SF** then assumes, first, a philosophically substantial distinction between general equations of motion (and the highly idealized models to which they apply) and instances of the equations of motion (and the less-idealized models to which they apply), and second the attribution to the former of a metaphysically privileged role. What this means is that, to some degree, idealizations do capture aspects of reality. That is, **SF** implies a form of *idealization realism* (IR). To put it differently, **SF** holds that symmetries are to be taken as metaphysically fundamental, but they are generally found in (highly) idealized laws.^10^ Then we are naturally committed to taking them as fundamentally real too. This is tantamount to endorsing IR.

No doubt that idealizations (as well as approximations and abstractions) play a central role not only in physics, but in science in general (see Fletcher et al., 2019). Regardless how useful they may be, idealizations are commonly viewed as “false” assumptions or “distortions” which are sources of certain “unfaithfulness” (Contessa, 2006) in scientific theories. One of the philosophical issues is that the extensive use of idealizations in science (in particular, in physics) seems to challenge any realist attitude towards covering laws, since they require *false* assumptions (e.g., perfect rigid bodies; see Cartwright, 1983; McMullin, 1985; Sorensen, 2012). To frame the issue correctly, idealizations have proved to be an extremely helpful tool to build a wide-ranging dynamics, to simplify and to handle complex physical problems more easily, but issues appear when we face the question of whether we should be realist about these wittingly introduced false assumptions on the base of its empirical and theoretical fruitfulness (Weisberg, 2007).

The challenge to IR can be posed in different ways. Two of them run as follows. Scientists employ idealizations (i.e., false assumptions) to explain and predict phenomena. By definition, false assumptions cannot be true, so they are “counter-productive in the pursuit of truth” (Sorensen, 2012: 30). Scientific realism, hence, implies that idealizations must be ineffective. However, idealizations are not only pervading, but also effective. Therefore, scientific realism fails and, with it, IR. An alternative way to see the challenge relates to whether the results of idealizations refer or not. Scientists employ false assumptions that lead to empirically successful science. If empirical success is a criterion for ontological commitments, then the realist ought to be committed to idealizations. However, if this is so, the realist ought to be committed to false assumptions. This discourages to take empirical success as the only criterion for ontological commitments, which undermines one of the main pro-realist arguments and the main support of IR.^11^

We can then wonder whether it makes sense to endorse IR to any degree. Note that if IR is rejected, and **SF** entails IR, **SF** must also be rejected by modus tollens. Then, it is a necessary condition to adopt **SF** to also adopt IR. And to adopt IR means that we should find the way to juggle realism with idealizations as false assumptions. Different way outs to this challenge have been proposed, and though they will depend on what kind of idealization we are assessing (e.g., Galilean idealizations, infinite idealization, multi-model idealizations, etc., see Weisberg, 2007), two general answers can be distinguished.

The first is to accept the indispensable role that idealizations play in scientific theorizing, but without placing them within our ontological commitments. Science is not only in the business of pursuing truth, but also in the business of achieving mathematical tractability, simplicity, predictions, effective control, and so on. Idealizations are indispensable to reach these aims and should be then accepted (this would amount to taking idealizations as *epistemically* indispensable). Thus, idealizations can be manageable in a scientific realist context, without the commitment that they yield laws (and models) that “latch onto reality”. However, it is easy to see that this answer is unsatisfactory for IR and, consequently, for **SF**. What it is needed is not to accommodate idealizations in a scientific realist framework, but to construe idealizations as *literally* true, that is, as truth-conducive devices or reference bearers.

The second answer tries to accommodate idealizations as literally true.^12^ Juha Saatsi (2016) has argued that there is a sense in which idealizations yield (covering) laws that do “get things right”. To account for the empirical success of idealizations, the realist ought to accept, and explain further, that idealizations do “get things right” by latching onto an unobservable reality responsible for their empirical success. This would juggle “falsehoods” with realism in a robust sense. According to him, idealizations yield structures that present us with *modal information* which is crucial for prediction. The important aspect of these structures is that they do contain false assumptions (e.g., a classical system in a perfectly homogeneous potential), but also veridical assumptions about the target. Whereas variation in the false assumptions does not undermine prediction, the “contained” veridical assumptions “can be viewed as latching onto reality so as to ensure the model’s predictive success” (2016: 10). In the end, falsehood is not an issue for IR, since it indeed captures some modal truth out there in the world. But, on which do these modal truths depend? For Saatsi, they are underwritten by real laws of nature. He says: “given these laws, from a scientific point of view, such a property [referring to a property obtained in the idealized model] can be a genuine, bona fide feature of the world on which our theorizing can latch” (2016: 13).

In saving IR, the inference from **SF** to IR is also saved. Yet, it is left to analysis which are the implications of endorsing IR. What Saatsi’s reply suggests is that idealizations latch onto reality because they ultimately capture modal patterns otherwise unreachable. If this is so, that idealizations are literally true and do get things right requires the *reification* of further theoretical structures that idealizations help us to capture and, in virtue of which, they turn out literally true—after all, the laws do not lie (pace Cartwright). This means that IR in turn entails to take further theoretical structures as metaphysically fundamental too, which amounts to introducing in one’s fundamental ontology such theoretical structures as the bearers of the symmetries. Indeed, intuitively, the idea looks quite natural: symmetries are not floating in the air, but they appear in the formalism of a physical theory as properties of mathematical structures (see McKenzie, 2014: 1101). Hence, it is reasonable that if symmetries are to be taken as metaphysically fundamental, those structures are to be also taken as metaphysically fundamental.

But which are those structures? I have said that symmetries are typically properties of equations of motion. A straightforward interpretation is to claim that those mathematical structures *stand for* laws of nature, that is, modal relations in the world that govern the phenomena (see Armstrong, 1983 for the *governing* view of laws; see Maudlin, 2007 for the *productive* view of laws). **SF** would then entail that symmetries are aspects of reality because they are properties of modal nomological structures that govern (or produce) phenomena. A similar view can be also found in ontic structural realism. It defends structures as ontologically fundamental, while entities or properties as metaphysically thin (see French, 2014: 178). These structures, in turn, include a primitive modality (Ladyman, 1998; Ladyman & Ross, 2007), which is expressed through the laws and the symmetries (French, 2014: ch. 10.2). An alternative would be to reify the geometrical structure (specially for geometric symmetries)—**SF** would then entail that the underlying geometric space (which instantiates the symmetries) is also part of the fundamental ontology. Different versions of space–time substantivalism (Hoefer, 1996; Maudlin, 1993), configuration-space realism (North, 2013) state-space substantivalism (Schroeren, 2020) or Hilbert-space realism (Albert, 1996; Carroll & Singh, 2018) may well serve to provide some metaphysical support to **SF**.

In the Introduction, I had advised that symmetries should not be analyzed in isolation, but rather in relation to a network of further ontological and epistemic commitments. Now the idea is clearer—**SF** requires IR which in turn requires the reification of further theoretical structures (e.g., endorsing realism about the dynamical structure). Then, the price to pay in endorsing **SF** is to also adopt a series of additional epistemic and ontological commitments—i.e., the existence of such structures at the fundamental level of reality and our epistemic, scientific access to them. Otherwise, **SF** remains unwarranted. We might at this point wonder whether it is a high price to pay. Of course, without any alternative on the table it might be way too soon, but I think it is already a red flag for those that are suspicious about adopting too many ontological commitments and a reason for pursuing an alternative. Indeed, to introduce, for instance, real, modal nomic relations in one’s ontology could be seen as overloading one’s ontology by Humeanism. Or, rather, to adopt substantivalism about space–time (or state-space) could be seen as unnecessary from a relationalist viewpoint. The point I want to make is that **SF** does not come for free and that the surplus price we are to pay for it might turn out too high for many. Whether the price is too high is a metaphysical issue, which should be assessed metaphysically. Those who are relationalist with respect to the geometric structure, or deflationist with respect to the dynamical structure (various versions of Humeanism, or dispositionalism for instance) do not have in fact many reasons to adopt **SF**. If they can somehow make sense of **the symmetry fact** and their successfulness without overloading their ontologies, then they will probably see **SF** as a too expensive proposal. If parsimony rules ontological commitments, then the search for an alternative view that does not overload one’s ontology seems to be worthy.

However, independently from whether such an alternative can be provided, to place **SF** within a broader network of ontological commitments opens the door to the obvious counterargument: if, for whatever reason, we reject any reification of theoretical structures, IR goes away. With it, **SF** lacks support. The counterargument is a modus tollens—if **SF** entails IR, and IR entails, for instance, the reification of the dynamical structure, then the rejection of this reification implies the rejection of IR and **SF** as well. Alternatively, one could reject **SF** on the basis of its commitments to take idealizations metaphysically seriously. In one way or another, this shifts the discussion from symmetries to realism about other theoretical structures. Therefore, **SF** ultimately depends on adopting realism about those structures. My point is, once again, that whether we should accept or reject these additional commitments is a metaphysical issue, assessed according to the standards of debates in metaphysics. This may now look trivial, but it seems to me that it has not been stressed enough in the current literature. If **SF** makes sense only when embedded in a network that adopts the appropriate ontological commitments to justify it, then everything must be assessed jointly and not in isolation.

### Second Problem. *Stipulations vs. Realism*

In the previous section, I arrived at the conclusion that covering laws (or highly idealized models) are those that generally exhibit the symmetries of philosophical interest. Now we can wonder how such symmetries became established in the first place. Did physicists discover them? Or did they rather stipulate them when formulating physical theories? I want to draw the attention to a sometimes-overlooked distinction between two approaches to symmetries—*by-stipulation* and *by-discovery*. Whereas the former takes symmetries as postulates, being prior to the details of the dynamics, the latter takes symmetries as a result of the details of the dynamics. In other words, by-stipulation symmetries *constrain* the dynamics since they act as rule-prescribing theoretical principles for formulating the dynamics of the theory. By-discovery symmetries rather *depend* on the structural relations already settled by the dynamics, revealing pre-existing features (see Redhead, 1975; Lange, 2007; Brading & Castellani, 2007 for similar distinctions). I claim that if the by-stipulation approach to symmetries is to be taken, then strong realist commitments to symmetries are under threat. With it, **SF**. It happens that, for some symmetries of interest as space–time symmetries and gauge symmetries, the by-stipulation approach turns out to be the common view in physics theorizing.

To illustrate this contrast, let me provide some examples. Detlef Dürr and Stefan Teufel, in laying the groundwork for Bohmian Mechanics, write:

“A symmetry can be a priori, i.e., the physical law is built in such a way that it respects that particular symmetry by construction. This is exemplified by spacetime symmetries, because spacetime is the theater in which the physical law acts (as long as spacetime is not subject to a law itself, as in general relativity, which we exclude from our considerations here), and must therefore respect the rules of the theater”. (2009: 43–44)

Since we impose space–time symmetries on the general laws to adapt them to some pre-existent spatial–temporal rules, this is a case of the by-stipulation approach. A similar point is raised by Robert Sachs (1987) in accounting for time-reversal invariance in physics. He says that.

“In order to express explicitly the independence between the kinematics and the nature of forces, we *require* that the transformations leave the equations of motion invariant when all forces or interactions vanish” (Sachs, 1987: 7. Italics mine).

Another clear case is the association of gauge symmetries with Yang-Mills gauge theories of particle interactions. Consider phase transformation ($\psi \to {\psi }^{i\alpha }$) for the simplest non-relativistic Schrödinger field ($\psi (x)$). Since the $\alpha$ factor does not depend on coordinates, it is a global symmetry transformation. But we could “localize” the transformation by making it dependent on spatial coordinates ($\alpha (x)$ for one dimension). In this case, local gauge invariance is imposed on the theory and on the interactions it describes. Yang and Mills’ seminal paper discusses the possibility of SU(2) local field theory. They assert that.

“We wish to explore the possibility of *requiring* all interactions to be gauge invariant under *independent* rotations of the isotopic spin at all spacetime points, so that they relative orientation of the isotopic spin at two spacetime points becomes a physically meaningless quantity” (Yang & Mills, 1954: 192. Italics on “requiring” are mine. Otherwise, original italics)

Contrast these cases with others that can be also found in the literature. Joseph Lagrange (1811) wrote that symmetries and conservation laws “must be viewed as general results of the laws of dynamics rather than fundamental principles of this science” (Lagrange, 1811: 241). A similar approach had been adopted by Isaac Newton in formulating classical mechanics in the *Principia* as the relativity principle appears as a corollary of the equations of motion (Newton (1729) [1687]: Book 1, Corollary VI. See also Lorentz and Poincaré for similar views. Brading & Castellani, 2003: 6). Thus, in these cases, symmetries seem to be discovered after the (classical) dynamics has been provided.

John Earman (2004) also notes two approaches, although he describes them a bit differently. He says that

“The received wisdom about the status of symmetry principles has it that one must confront a choice between the *a posteriori approach* (a.k.a. the bottom-up approach) versus the *a priori approach* (a.k.a. the top-down approach)”. (2004: 1230. Italics in the original)

Interestingly, Earman (1989) also mentions that symmetry principles are frequently considered contingent, rather than necessary. In discussing active and passive symmetry transformations in relationalist and substantivalist frameworks, Earman claims that the relationalist is committed to a passive reading, where the symmetry transformation connects different descriptions, being all of them equally accurate. But, if this is so, then:

“it would seem that the symmetry transformation could not fail to be a true symmetry of nature, contradicting the usual understanding that symmetry principles are contingent, that is, are true (or false) without being necessarily true (or false)”. (1989: 121)

Certainly, whereas the idea of stipulating a symmetry suggests certain degree of “normative necessity” for some aspects of a physical theory, the by-discovery view, once we have found empirically successful laws, suggests that we can extract the symmetries from the laws by investigating their empirical and theoretical implications. Although the by-discovery approach was much more common in the nineteenth century, the “reversal of the trend” seems to have come with the formulation of special and general relativity (see Wigner, 1967) and with the raise of the gauge paradigm—from a “descriptive view”, where symmetries are seen as something-to-be-found in the laws (discovered in the laws), to a more “normative” one, where symmetries are stipulated in order to shape the laws.

Having laid out the general features of both approaches, I offer two more specific definitions.

**By-stipulation**: A symmetry is to be regarded as a guiding principle that is **a priori** and **T-NEC** (“necessary for the theory T”).
**By-discovery**: A symmetry is to be regarded as a theory consequence that is **a posteriori** and **T-CON** (“contingent for the theory T”).

Both approaches seek to explain how **the symmetry fact** comes about. Let me explain these definitions a bit further. The epistemic notions of “being a priori/posteriori” are to be understood in relation to whether a symmetry is known independently from the dynamics’ details and to whether it is a condition to obtain (or derive) a dynamics (see Redhead, 1975). The notions of “being necessary/contingent” refer to *physical* necessity and to whether it is possible for an approximately true physical theory to lack a symmetry or not. That’s why necessity/contingency need to be relativized to a physical theory (T-CON/T-NEC). The distinction basically seeks to capture whether it is possible for a physical theory to remain the same theory but exhibiting different symmetries (e.g., failing to exhibit certain symmetries).^13^

Despite the difference between **SF** and **SInd**, **SF** seeks to make justice to the indispensability of **the symmetry fact** by holding that we ought to take it as metaphysically fundamental—symmetries as part of the common minimal basis of entities, properties or relation that determines everything else. If I am right and there are indeed two different approaches to elucidate how **the symmetry fact** comes about in a physical theory, it is worth investigating whether **SF** can accommodate either approach. It is easy to see that **SF** can easily accommodate the by-discovery view. As **the symmetry fact** is an aspect of fundamental reality, it may express, for instance, facts about the properties of modal structures or patterns which come to be discovered through scientific modelling, laws of nature, and idealizations. It just happens that reality comes fundamentally equipped with some symmetries as it also comes fundamentally equipped with, for instance, some modally robust patterns. Symmetries are just fundamental properties of those structures in our ontology, and we discover the symmetries as we discover such modal patterns. In other words, we intend to capture such symmetries in the ontology through the mathematical representation of the natural world as we intend to capture modal patterns by introducing dynamical equations in our physical theories. Idealizations are truth-conducive processes after all, and symmetries are properties of such idealization products.

It is noteworthy that, according to the by-discovery approach, there is an epistemic gain about the natural world in knowing laws’ and/or geometry’s symmetries, since we have independent reasons to have the dynamics and the geometry as we have them–our theories need not to have the symmetries they actually have, because they might fail to capture them (as many physical theories have failed to capture the relevant regularities by introducing the wrong laws). As the scientific enterprise discovers nomological relations held among phenomena, it also discovers symmetries in such nomological relations—physics presents us with **the symmetry fact** because the putative references to symmetries are true in virtue of facts in the world about symmetries: Facts related to the common minimal basis. The by-discovery view of symmetries and **SF** then naturally articulate each other. They render a view conforming to which symmetries mainly play a descriptive role, in physics as well as in metaphysics—a symmetry statement tells us what the world is ultimately like.

Yet, **SF** no longer looks so persuasive when it comes to the by-stipulation approach. The tension is between the *descriptive* or *representational* nature of **SF** and the rather *normative* import of the by-stipulation approach. Note that the conflict does not arise because symmetries are merely stipulated, but because the stipulation of symmetries is meant to impose a normative constraint over theory construction. Hence, there seems to be a conceptual conflict between taking putative references in **the symmetry fact** as referring to fundamental aspects of reality and taking the as a normative principles guiding theory construction. This tension and conceptual conflict can be illustrated in terms of the distinction between the context of discovery and the context of justification (see Schickore, 2018).

Even though there has been ample discussion about the distinction between context of discovery and of justification (see, for instance, Leplin, 1987; Weber, 2005), it is generally interpreted as involving two different epistemic processes—that of conceiving a theory and that of validating it. When symmetries are regarded as capturing the deep structure of the world (revealing the “logic of nature”), a physical theory gains epistemic support and semantic content. In this sense, symmetries play a role in validating a physical theory within the context of justification. But, when symmetries are placed as constraints or metalaws that regulate and dictate the development of the dynamics, symmetries play a role in conceiving a theory, that is, play a role within the context of discovery. The tension here is that **SF** and the by-stipulation approach place symmetries in two different contexts in the enterprise of conceiving, formulating, and validating a physical theory.

Consider the demand of local gauge invariance in the Standard Model. On the one hand, they are supposed to reveal deep features of reality since they “dictate” the interactions that govern phenomena and the entities (particles/fields) that exist at the fundamental level. On the other hand, the demand of local gauge invariance seems to rely on a very pragmatic basis, being divorced from deep ontological considerations. Christopher Martin (2002, 2003) has argued that this demand “might seem to constrain any real physical import of gauge principles to the context of discovery” (2003: 41). He adds that then “we should then count ourselves amazingly fortunate that the “right” theories just happened to have such a nice structure, i.e., that seen in the theories’ tight group-theoretic structure which accompanies the characteristic symmetry/invariance” (2003: 41). However, **SF** goes beyond the context of discovery by invoking symmetries in the context of justification. This is the role of the so-called “gauge philosophy”, which takes local gauge symmetries as deep physical principles *because* reality itself obeys to such symmetry principles. This could easily be seen as a particular case of **SF** for gauge symmetries.

In placing symmetries as elements in the context of justification, as **SF** seems to be committed to, is not a problem per se, but it becomes one when combined with the by-stipulation approach, which suggests a completely different epistemic role for them. The tension is clear in their semantic aspect. **SF** is committed to affirm that symmetry claims (i.e., claims about **the symmetry fact**) are true or false. Stipulated symmetries, nonetheless, are not the right sort of statements that are true or false, but that are meant to play various roles. They may be introduced for the sake of systematization, simplification, unification, interconnectedness, mathematical and physical tractability, among other non-empirical virtues. Even worse, some elementary symmetries (e.g., space-translation invariance) seem to be not even open to empirical revision. The reasons are that many elements that intervene in theory construction do not need to latch onto the ontology, nor play any referential role, but they are mostly guided by more principled reasons and relate to the very conditions of acceptability of a physical theory. Most symmetries of philosophers’ interest, it seems to me, are of that kind. The last judge is not ultimately reality, but the epistemic and normative background underlying theory construction and modelling.

Which role by-stipulation symmetries may play will depend on the philosophical background. Pragmatists could view them as mere tools or conventions (as linguistic rules), that are clearly not false or truth, but useless or useful (see Richman, 1961). Of course, rules are non-arbitrary and could demand a very complex epistemic process, following in general various demands for their acceptability; in any case, truth is not one of them, at least, not immediately or alone. But symmetries could have a rather more crucial role. Neo-Kantians, for instance, may view symmetries as either regulative or constitutive principles that set the conditions for physical objectivity (see Cassirer 1923, Heis, 2014). More specifically, symmetries are a priori *norms* that are the transcendental conditions of objectivity in modern physics, since they impose independence of some features from variations of perspective and/or initial conditions. A good example of this is the wide range of spatial and temporal symmetries, which constitute the idea of objectivity as perspective-independent, that is, independent from spatial and temporal location or orientation. Likewise, the requirement that the laws be formulated in a covariant way was crucial for some Neo-Kantians in the early twentieth century since covariance sets the conditions for a proposition to be physically meaningful (see Ryckman 2008 and references to Hilbert’s work therein). Eugene Wigner (1949, 1963), for instance, held that symmetries are the principles that make science possible, in the sense of being the principles that make laws possible (Wigner 1949: 521). It is clear, in this last case, that those principles are not either true or false, but they are normative in a transcendental sense.

These are not the only ways in which by-stipulation symmetries can be construed, but, be that as it may, the burden is now on the **SF**’s side: she ought to argue in which sense symmetries can be true or false and, at the same time, be normative stipulations in the process of theory construction; or, alternatively, argue why they seem to be normative, but they actually are not. It might be counter-argued that symmetries are part of a general theory’s empirical virtues, but they nonetheless contribute to truth-conduciveness: inductive evidence could suggest that physical theories that conform to such virtues are likely to be true. Then, symmetries may well be a normative constraint for the construction of future physical theories since it is reasonable, in the light of inductive evidence, to expect that future *true* physical theories exhibit some symmetries.^14^ This is a good argument, but I think it is not sufficient.

To begin, it would only prove that a mild form of symmetry realism could be true, but it is not sufficient for **SF**. Future physical theories should be ontologically committed to symmetries (according to this argument), but it does not imply that future physical theories should take them as ontologically *fundamental*. An additional premise is needed. Second, physical theories turn out to be true or false as a whole. There are good reasons to suppose that some form of the Quinean semantic holism is true, so it is difficult to claim that because a physical theory is true (which here means basically “empirically adequate”^15^), then a specific element of it is responsible of its (approximate) truth. Here again, an additional argument is needed: as physical theories face the “tribunal of experience” as a whole, it is necessary to say why this specific element is responsible for its success.

Third, the argument could be turned around: there is inductive evidence that most of the stipulated physical symmetries turned out to be strictly false. Not only are they violated in the majority of models of physical theories (remember the systems that have many complex non-conservative interactions), but also most physical symmetries of older theories have been replaced by the symmetries of newer, more successful theories. In any case, when ontological commitments to symmetries are taken, we step out of the context of discovery to step into the context of justification—they become valid not because they lead us to, for instance, better systematized theories or ground the concept of physical objectivity, but because they ultimately represent aspects of reality. If this is so, then all symmetries must be open to empirical revision. However, this seems to not be always the case.

In any case, even though the argument might escape the previous points, I still think that it needs to be complemented with the assumptions mentioned in Sect. 3.1. I do not mean to raise a knock-down argument against **SF**, but to say that it only makes sense if additional assumptions are taken; assumptions that can be reasonably rejected.

## Final Remarks and Future Work

This paper is meant to be a call for caution if **SF** is to be endorsed. I have backed this call for caution by raising two critical arguments to which **SF** ought to reply. The first argument centered on the role played by idealization to obtain most symmetries. I argued that** SF** entails IR, but to endorse IR does not come for free, since additional theoretical structures need to be reified and introduced in the fundamental ontology. Though this is not problematic per se, it might be undesirable from the perspective of ontological parsimony, and of philosophical frameworks that oppose such reifications (e.g., Humeanism about laws, relationalism about geometry, etc.). This argument, in addition, placed symmetries within a broader network, where our view on them also depends on our views on laws of nature, or the nature of the underlying space. This calls for also assessing **SF** in a broader metaphysical landscape and according to metaphysical standards.

The second argument centered on the distinction between two approaches to symmetries (by-stipulation and by-discovery) and showed that endorsing **SF** and the by-stipulation approach is troublesome. The main issue is the conflicting roles assigned to symmetries: where **SF** regards symmetries in physics as descriptive statements about the fundamental reality, the by-stipulation approach regards them as rule-prescribing principles that play a normative role in theory construction and empirical research. I illustrated this opposition by invoking the distinction between the context of justification and the context of discovery.

To conclude, let me briefly expose some lines of future exploration. One of the most appealing aspects of **SF** is that it makes justice to the putative references to symmetries in **the symmetry fact** and its successfulness: symmetries do seem to be indispensable in physics (**SInd**). Does the rejection of **SF** entail that we should also reject **SInd**? I think it does not. To begin, we may remove the idea that symmetries are fundamental, which leaves us with some form of weaker realism. Yet, I think this is an unattractive position—after all, symmetries would be part of the derivative ontology, so it would not be so clear why they are indispensable but real. A more interesting alternative is to distinguish, within **SInd**, between “ontologically indispensable” and “epistemically indispensable”. This division would allow us to retain **SInd** while avoiding some form of mere conventionalism. Further work needs to be done, but a plausible strategy is to defend some form of *symmetry deflationism*, where symmetries are indispensable (endorsing, thereby, **SInd**), but as epistemic principles in theory construction and/or in understanding the physical world. For instance, Humeanists that also endorse the Best System Approach to lawhood may also endorse some form of symmetry deflationism by placing symmetries as indispensable meta-laws in the best system. Or, Neo-Kantians may construe symmetries as underlying the conditions of physical objectivity and, a fortiori, the conditions for having physical laws as we have them.

As I said repeatedly, I did not offer any knock-down argument against **SF**. Nor did I prove it wrong. But, as mentioned in the Introduction and along the paper, I meant to call for some philosophical caution. I tried to do this by placing **SF** in a broader network of philosophical commitments and in exploring some of its consequences. These caveats, I trust, justify pursuing alternative approaches.

## Data Availability

Not applicable.

## Footnotes

1 I am not adopting a model-set-theoretic view of models here (Earman 2004; Suppe 1989; van Fraassen 1989), but a more pedestrian one. I am thinking of models as conceptual objects whose entities and processes (more or less idealized) are described by a theory’s equation of motion (e.g., free-particle model, Lorentz gas model, etc.).

2 Eugene Wigner draws a distinction between *dynamical* symmetries and *geometrical* symmetries. But, whereas for Wigner geometrical symmetries coincide here with spatio-temporal symmetries (as well as with *global* symmetries), dynamical symmetries are *local* gauge symmetries (see Wigner 1967: 17). Hence, his notion of dynamical symmetries is different from other uses of the same notion, for instance, by Earman 1989 and Wallace 2019. I am here following Earman’s and Wallace’s meaning of dynamical symmetry, which mainly refers to the idea of a symmetry instantiated by a dynamical equation of motion.

3 This indispensability argument does not say that symmetries ought to be taken as real *qua* mathematical entities (as the original indispensability argument would do), but as physical ones. An additional argument should be then provided to justify such a jump (see Matarese 2022).

4 After all, if some theoretical posits are introduced in one’s ontology as an ontological free lunch, then one’s ontology is no less sparse for containing them than it is for containing the entities which ground them. Even though this does not strictly mean we can dispense with the derivative ontology, the type of Quine-Putnam Indispensability Argument I am evoking here seems to claim that symmetries are the sort of ontological posits that ground everything else.

5 Indeed, I have drawn the name ‘symmetry fundamentalism’ from his work.

6 In her new book, Jill North (2021) claims: “There is a reason for formulating things in terms of structure rather than symmetries, though. Structure is what we are ultimately after (both mathematical structure in the formalism and physical structure in the world), and symmetries are simply an (important) guide to that structure. As mentioned in Chapter 2, symmetries are an indicator of structure, not the structure itself. More importantly, there can be more to the requisite structure than what seems to be indicated by dynamical symmetries” (2021:73).

7 It is important to note that in a fundamentally non-time-reversal invariant world, the postulation of very special initial conditions (i.e., a Past Hypothesis) is no longer necessary to account for temporal asymmetries.

8 For instance, consider the canonical time-reversal transformation in Newtonian classical mechanics. The Newton Second Law is said to be invariant under time reversal. However, as Hutchison 1993 put it, it will mostly depend on the intervening forces. An instantiation of the Newton Second Law where non-conservative forces intervene (e.g., friction), it will be non-time-reversal invariant.

9 The term idealization is a rather vague one. Technically, there would be at least three notions that, though closely related, we should keep conceptually apart: idealization, approximation and abstraction. Patrick Suppe (1989) has distinguished between idealizations and abstractions (for details, see Suppe 1989: 82-83, 94-99). John Norton (2012) has in turn distinguished between idealizations and approximations. Though I acknowledge the importance of the distinctions and the nuances each involves, I will use the term ‘idealization’ as an umbrella term to refer to any theoretical activity that involves the introduction of distortions, simplifications or abstractions (see McMullin 1985: 248).

10 There might be an ambiguity here. One thing is to say that symmetries *are* idealizations (see Oldofredi and Öttinger 2021, for instance). Another thing is to say that symmetries are *properties of* idealization products. I might well endorse both views, but I’m now focusing on the latter.

11 This is, of course, an instance of the common resistance to the non-miracle argument –empirical success does not entail truth-conduciveness (see van Fraassen 1980, Laudan 1981).

12 Michael Strevens (2011) tries to reconcile idealizations with causal explanations or causal histories. However, it is not so clear that he is defending some strong realist reading of idealizations, rather than showing that they are essential to get some explanations right (e.g., by ignoring some irrelevant details). **SF** must take idealizations metaphysically seriously as they belong to the fundamental ontology. I am not sure if Strevens’ argument in favor of the truth-conduciveness of idealizations (in some cases) is sufficient for **SF** because it is not clear to me which ontological stance Strevens takes towards idealizations, beyond the compatibility with realism.

13 Consider the following example. Non-relativistic standard quantum mechanics is Galilei invariant. Now, must it be Galilei invariant? Or does it just happen that it is Galilei invariant? When assessing alternative quantum theories, the question becomes relevant. Alternative quantum theories are expected to, at least, recover the standard quantum observable predictions, but should they also recover its symmetries? Some have, for instance, argued that wave-function realism fails because it is not Galilei invariant (see, for instance, Allori 2017). This criticism, I claim, supposes a by-stipulation view of symmetries –non-relativistic quantum mechanics must be Galilei invariant, so any alternative theory that cannot accomplishe Galilei invariance flat-out loses. If symmetries are supposed to be discovered, then the argument no longer runs.

14 I thank an anonymous reviewer for raising this argument.

15 It is helpful to remember that empirically adequacy does not necessarily amount to truth, nor is it sufficient.
